# The effectiveness of additional screening examinations for children and adolescents in Germany: a longitudinal retrospective cohort study

**DOI:** 10.1186/s12887-023-03988-1

**Published:** 2023-04-11

**Authors:** Kathrin Krüger, Anne-Marie Lapstich, Katrin Christiane Reber, Stephanie Sehlen, Sebastian Liersch, Christian Krauth

**Affiliations:** 1 Institute for Epidemiology, Social Medicine and Health Systems Research, Hannover Medical School, Carl-Neuberg-Str. 1, 30625 Hanover, Germany; 2 Center for Health Economics Research Hannover (CHERH), Otto-Brenner-Straße 7, 30159 Hanover, Germany; 3 AOK Nordost. Die Gesundheitskasse, Health Services Management, Wilhelmstr. 1, 10963 Berlin, Germany

**Keywords:** Prevention, Early detection, children, Adolescents, Routine data, Statutory health insurance data, Germany

## Abstract

**Background:**

Continuous medical care is particularly important in childhood and adolescence. Since there are gaps in regular care in Germany, various health insurance providers offer to cover additional examinations (e.g., U10, U11, J2) to ensure ongoing paediatrician visits. However, the question arises as to whether these examinations are effective. Thus, the main objective of this study is to determine whether participation in the U10, U11 or J2 examinations leads to more frequent and earlier diagnosis and treatment of age-specific diseases.

**Methods:**

The analyses are based on administrative claims data from a statutory health insurance fund. For each examination, an intervention group (IG) is formed and matched with a corresponding control group (CG). Descriptive analyses include proportion with diagnosis and treatment, average age of diagnosis and treatment initiation. Hypothesis testing is performed using methods appropriate to each. In addition, subgroup analyses and binominal logistic regression models are conducted.

**Results:**

More diagnoses are detected in IG, irrespective of subgroups. Additionally, diagnoses are made slightly earlier on average in IG. In the total samples, more therapies are initiated in IG, and slightly earlier. Considering only diagnosed cases, more therapies are initiated in CG but continue to be started earlier in IG. Regression models show that participation in the examinations has the highest predictive power for detecting a diagnosis. The presence of a chronic disease and sex - male at the U10 and U11 and female at the J2 - are also significantly associated. The models further show that nationality, unemployment of parents and region also have a significant influence in some cases, whereas school-leaving qualification, vocational qualification and income of parents do not. Considering the initiation of treatment in overall samples, the models show similar results, but here the presence of a chronic illness has the highest predictive power.

**Conclusion:**

The results indicate that participation in the examinations leads to significantly more diagnoses and, in the overall samples, significantly more treatments. In addition, diagnoses were made somewhat earlier and therapies were initiated somewhat earlier. In the future, it would be useful to investigate the U10, U11 and J2 examinations over a longer time horizon to determine whether the statistically significant difference found is also clinically relevant, i.e., earlier diagnosis and initiation of therapy lead to prevention of manifestation or progression of the diagnosed diseases and to avoidance of secondary diseases.

**Trial registration:**

German Clinical Trials Register (DRKS), DRKS-ID: DRKS00015280. Prospectively registered on 18 March 2019.

**Supplementary Information:**

The online version contains supplementary material available at 10.1186/s12887-023-03988-1.

## Background

**Fig. 1:**
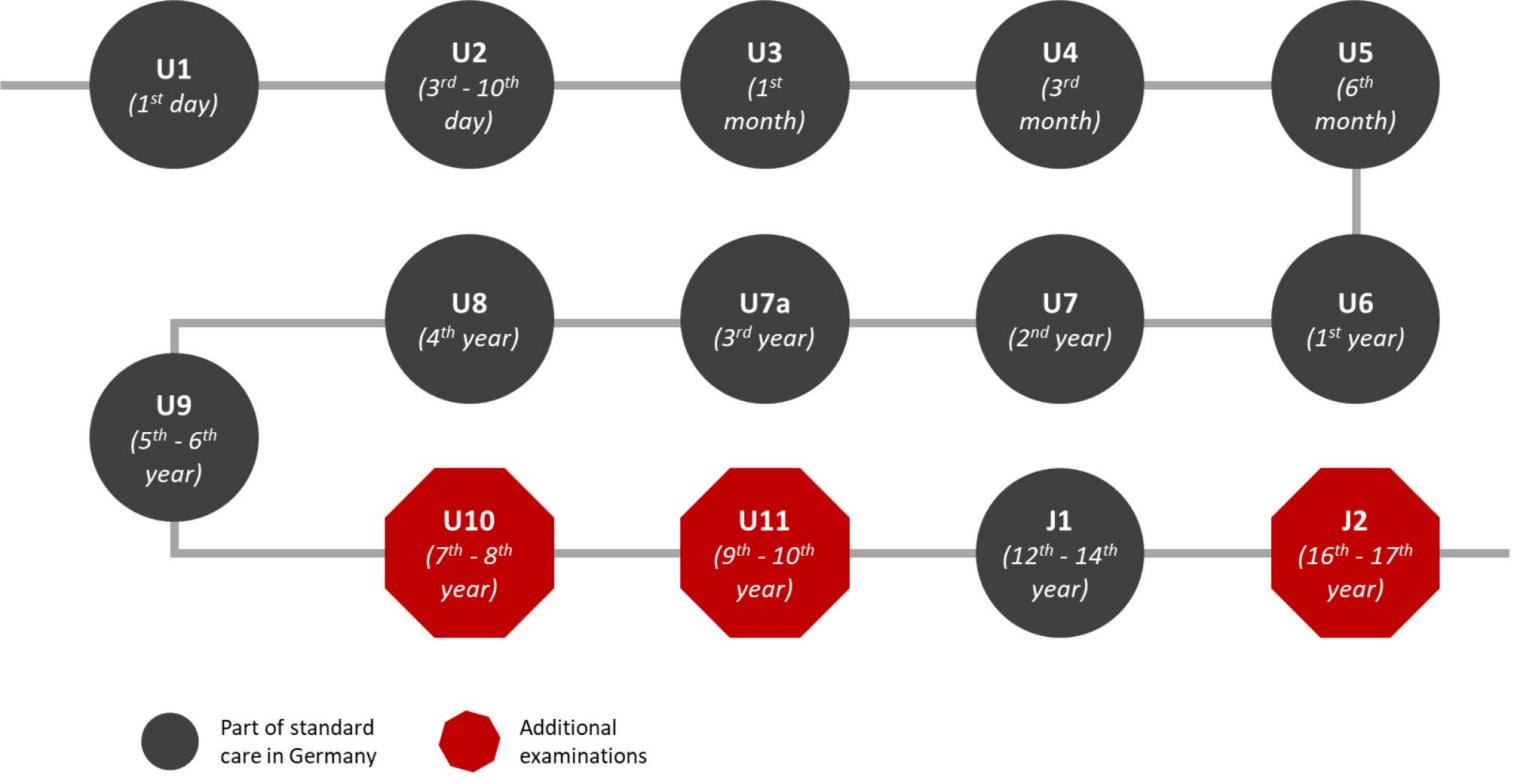
Preventive medical examinations for children and adolescents in Germany

Early detection tests can prevent the manifestation of diseases, mitigate consequences and prevent long-term health risks by detecting and treating early stages of diseases. Since diseases and their early stages develop at different ages, continuous care and regular examinations are particularly important in childhood and adolescence [1–3]. In Germany various age-specific health examinations (called U-Untersuchungen) for children and adolescents are defined as services provided by the statutory health insurance (currently U1 to U9 and J1). The contents, timing and structure of the individual examinations are defined by the Federal Joint Committee (G-BA) and consist of special preventive examinations for certain age-specific diseases as well as the physical examination of the child and counselling of the parents. All children and adolescents in the relevant age groups can take part in the examinations. However, there are no regular screening tests for children between the ages of 7 and 10 and for adolescents older than 15 as part of standard care provided by the statutory health insurance (see Fig. 1) [4]. In Germany, in addition to statutory health insurance, there is also private health insurance. The latter is aimed at all those who are not compulsorily insured under the statutory health insurance scheme, e.g. due to a high income or self-employment. The proportion of people with statutory health insurance is 92% of the population. Only services that are part of standard care are covered by the statutory health insurance. Against this background, various statutory health insurance companies intend to close the existing gap in provision by offering additional services, such as covering the costs of the additional U10 (between 7 and 8 years), U11 (between 9 and 10 years) and J2 (between 16 and 17 years) examinations, with the objective of providing continuous medical care throughout childhood and adolescence [5]. In this context, some of these statutory health insurance funds have set up programmes for children and adolescents that offer additional services besides U10, U11 and J2.

In 2007, the AOK Nordost, a regional health insurance provider in the federal states of Berlin, Brandenburg and Mecklenburg-Western Pomerania, developed the *AOK-Junior* program for the early detection of diseases in children and adolescents. Within the framework of this health programme, various additional modules for the early detection and treatment of diseases are offered at different ages. These include, for example, the cost coverage for the preventive examinations U10, U11 and J2 as well as for check-ups for skin and lung diseases and interventions for dental health. To participate in this health programme, children and adolescents enrol with their paediatrician and are informed and advised by the AOK Nordost and their paediatrician about the modules. The effectiveness of the ongoing program was examined as part of the project “Evaluation of the Pediatric-Centered Integrated Care *AOK-Junior* (EPIVA)” which started in 2018 [6].

Although the utilization of the examinations offered can close the gap in continuous treatment for children and adolescents, the question arises as to whether the examinations are effective. Thus, the aim of the present analysis is to investigate whether the three modules that had by far the highest participation rates of AOK Junior - the U10, U11 and J2 - are effective in terms of diagnosing and initiating treatment for disease. The examinations involve counselling of patients and parents, but the main component is screening for general health problems and age-specific diseases [7].

The **U10** examination is a preventive screening for children of primary school age intended to take place between the ages of 7 and 8. Special attention is paid to developmental and behavioural disorders that often become apparent only after children start school, e.g. attention deficit hyperactivity disorder (ADHD), dyslexia, dyscalculia, and motor development disorders. The paediatrician will conduct the following tests with the child to diagnose general health problems as well as the abovementioned disorders: measurement of height, weight and blood pressure, general physical examination with assessment of organ function by listening and palpation, analysis of a urine sample, hearing and vision test and examination of the heart rhythm by means of an electrocardiogram. Additionally, the parents are asked to assess their child’s behaviour at school via a questionnaire [3, 8, 9].

The **U11** examination is primarily used to determine school performance and behavioural problems as well as difficulties in social interaction at the age of 9 to 10. For this purpose, the paediatrician conducts a number of tests with the child that focus on text comprehension and the ability to concentrate. In addition, a general health check takes place, similar to the U10 examination. In a discussion with the child and the parents, the paediatrician tries to determine how the child deals with stress and conflict situations. If the child does not go to the dentist regularly, the paediatrician also carefully examines the teeth and their position in the jaw. Another major topic of the U11 examination is advice on media use, substance abuse, nutrition and physical exercise [3, 8, 9].

The **J2** screening is the last before reaching adulthood and is recommended between the ages of 16 and 17. It is especially important to close the gap between the first youth health examination J1 at the age of 13 and adulthood due to the intense body changes in the middle of puberty. This is why puberty and sexuality disorders are among the main foci of the screening. Due to the growth spurt during puberty, many malpositions and deformities become apparent. Therefore, the skeletal system is examined particularly carefully. The physician pays special attention to postural weaknesses and pathological curvatures of the spine. As part of the examination, the physician also examines the thyroid gland by palpation, ultrasound and blood tests, and the family history is also taken into account. Another focus of the examination is the general state of health, especially obesity and nutrition, as well as the consequences of misbehaviour in this regard, which is why tests are also carried out for diabetes mellitus type II [3, 8–10].

In the context of these examinations, it is important to analyse whether they lead to earlier diagnosis of diseases and consequently to earlier initiation of therapies. Previous analyses of these early diagnosis examinations have mainly focused on participation rates and behaviour. Here, a social gradient is partly evident [11–15]. For this reason, it will also be investigated whether the generated results apply to all subgroups irrespective of education and income among other things. However, there is limited evidence for the effectiveness of screening examinations for children and adolescents [16]. Thus, this analysis is intended to contribute to the creation of an evidence base for screening programmes in children and adolescents.

## Methods

### Aim, design and setting of the study

The evaluation project EPIVA should provide information on the quality of *AOK-Junior* to derive recommendations for further development of the care model. In the context of outcome quality, an important question of the project is what effects the *AOK-Junior* programme has on the participants. To investigate this, the following four hypotheses are developed:

- **H**_**1**_: More diseases are detected in the intervention group (IG) than in the control group (CG).
- **H**_**2**_: Diseases are detected earlier in the IG than CG.
- **H**_**3**_: Early detection of disease in the IG leads to more therapies than in the CG.
- **H**_**4**_: Early detection of disease in the IG leads to earlier therapy than in the CG.

This study aims to answer these questions for the U10, U11, and J2 screenings using administrative claims data, i.e. the accounting data of the AOK Nordost, in a longitudinal retrospective cohort study. The programme cannot be evaluated in a randomized controlled trial because it has been running since 2007 and participation is voluntary. However, for the hypotheses investigated, the analysis of claims data seems to be practicable in many respects: (1) The outcome quality can be assessed on the basis of the incidence rate (see H_1_) and the time of detection (see H_2_) of a chronic disease. (2) A large number of cases can be examined. The IG is an almost complete survey. (3) The CG can be generated by individual matching.

All methods are carried out in accordance with relevant guidelines and regulations.

### Participants and material

All children or adolescents enrolled in the *AOK-Junior* programme and participating in one of the three screenings are allocated to IG. For CG, children and adolescents who are also insured with AOK Nordost but did not participate in *AOK-Junior* and did not take part in the screenings are selected. To analyse the effectiveness, one IG and one CG are defined for each of the U10, U11 and J2. A pre-observation period of 2 years and a post-observation period of 2 years are applied to all intervention and control groups. The index event for IG is the quarter in which the respective screening was carried out, and for CG, a quarter with a paediatrician visit at the same age is selected. Annual quarters are chosen because the claims data for outpatient care are divided into quarters due to the quarterly accounting in this sector. Both groups are specified as having none of the diagnoses defined for the respective screenings (U10, U11, J2) during the two-year pre-observation period, so that, as far as possible, only incident cases are considered in the analyses. IG and CG are matched in a 1:1 ratio using the following parameters:

- Sex (male/female).
- Age (U10: 7/8 years; U11: 9/10 years; J2: 16/17 years).
- Federal State (Berlin/Brandenburg/Mecklenburg-Western Pomerania).
- Presence of a chronic disease (yes/no).

The routine data of the AOK Nordost for the period 2010 to 2019 serves as the data basis for IG and CG. Data sets on outpatient services, inpatient services, prescription drugs, medical remedies, medical aids, and rehabilitation as well as basic data are included in the analyses. The data are subjected to a plausibility check prior to the start of the analysis.

### Definition of outcomes

Based on the contents of the individual examinations, various diseases and the associated ICD-10-GM codes (International Statistical Classification of Diseases and Related Health Problems, German Modification) are defined as outcomes; these are shown in Table 1. If the diagnosis was made in the outpatient sector, an assured diagnosis had to be coded by the physician. For the inpatient sector and rehabilitation, one of the defined diagnoses had to be coded as the main diagnosis. Concerning medical services, those that were coded along with one of the defined diagnoses and represented a disease-specific treatment were included. For pharmaceuticals as well as medical aids and remedies, the prescription has to be relevant for the respective coded illness and have a temporal connection with the diagnosis, i.e. the coding had to have taken place in the same (annual) quarter.

**Table 1:** Overview of included diagnoses and corresponding ICD-10 codes

	Diagnoses	ICD-Codes
**U10**	Disorders of psychological development	F80-F89
	Behavioural and emotional disorders	F90-F98
**U11**	Mental and behavioural disorders due to multiple drug use and use of other psychoactive substances	F19.0-F19.2
	Eating disorders and obesity	F50; E66
	Disorders of psychological development	F80-F89
	Behavioural and emotional disorders	F90-F98
	Reaction to severe stress and possible consequences (somatoform disorders and problems related to life-management difficulty)	F43.2; F45.3-F45.4; F45.8; Z73
	Diseases of oral cavity, salivary glands and jaws	K00-K14
**J2**	Disorders of thyroid gland	E00-E07
	Diabetes mellitus	E11-E14
	Disorders of puberty	E30
	Sexual dysfunction, gender identity disorders, disorders of sexual preference	F52; F64; F65
	Kyphosis and lordosis, scoliosis, other deforming dorsopathies	M40; M41; M43

For H_1_, the dependent variable is the proportion of children diagnosed during the post-observation period. Regarding H_2_, the dependent variable is defined as the period of time between the index quarter and the subsequent first diagnosis during the post-observation period, which is operationalised by the age at first diagnosis. For H_3_ the dependent variable is the proportion of children treated during the post-observation period. With regard to H_4_, the dependent variable is the period of time between the quarter of the first diagnosis and the quarter of the first relevant treatment, operationalised by the age at therapy initiation.

### Definition of determinants

In addition to participation in the respective examination (yes/no), additional determinants from the basic data of the AOK Nordost are used to investigate factors influencing the detection of a diagnosis and the initiation of treatment. The basic data contain information on annual gross income, school-leaving qualification and vocational qualification for the employed population as well as information on insurance and occupational status, nationality and region of residence. Some of the basic data are available only for the directly insured person (normally father or mother), since the children or adolescents are co-insured through them, and some data are available for the children or adolescents directly.

In terms of school-leaving qualifications of the directly insured person, three categories are formed: (1) no school-leaving qualification and a low-level qualification equivalent to 9 years of schooling were defended as low, (2) an intermediate qualification equivalent to 10 years of schooling corresponded to middle and (3) a qualification in the form of a school graduation certificate or technical diploma equivalent to 12 or 13 years of schooling equalled high. In terms of vocational qualifications of the directly insured person, the following groups are formed: (1) no qualification, (2) completed vocational training and (3) higher degrees from universities and other higher education establishments. In Germany, it is mandatory to be insured in the statutory health insurance, but above a certain income threshold it is possible to be insured in the private health insurance scheme or to be voluntarily insured in the statutory health insurance. Therefore, the insurance/occupational status has been divided into compulsorily insured and voluntarily insured, and there are also the categories of unemployed and other. The latter includes pensioners, rehabilitants and family members. In terms of income of the directly insured person, the insured are divided into three groups: low (less than 70% of median income), medium (70–150% of median income) and high (over 150% of median income) [17]. The nationality is divided into two groups, individuals with German nationality and individuals with a nationality other than German. Based on the classification of Eurostat, the following division is made for the region of residence: predominantly urban regions, intermediate regions and predominantly rural regions [18].

### Statistical analysis

In hypotheses H_1_ and H_3,_ both the outcome (H_1_: diagnosis (yes/no); H_3_: therapy (yes/no)) and the determinant (participation in an examination) are nominally scaled. Therefore, Fisher’s exact tests are calculated to test both hypotheses. In hypotheses H_2_ and H_4_, the outcome (H_2_: age at diagnosis; H_4_: age at therapy initiation) is metrically scaled, so Mann-Whitney U tests are used here to test the hypotheses. In addition, the detection rates are analysed using stratification according to different parameters to investigate differences between subgroups. These subgroup analyses are conducted with regard to school leaving qualification, vocational qualification, insurance/occupational status, income, nationality, and region using cross tables. To analyse differences, we conduct Chi-square and Fisher’s exact tests. Furthermore, binominal logistic regression modelling for H_1_ and H_3_ is performed to identify associations between characteristics of the children or adolescents and the diagnosis or the treatment of diseases for the U10, U11 and J2. Due to the heterogeneity of the diseases, binominal logistic regression models for individual diagnostic groups are also calculated for the U11 and J2. Regarding treatment initiation, binominal logistic regression models are calculated for the total population as well as for the group of diagnosed individuals. The following variables are first analysed and those shown to be associated with the outcome below p < 0.25 are included in the respective model: group *(CG (0); IG (1))*, sex *(female (0); male (1))*, chronic disease *(no (0); yes (1))*, school leaving qualification *(none or low (0); medium (1); high (2))*, vocational qualification *(none (0); completed vocational training (1); higher degrees (2))*, unemployment *(no (0); yes (1))*, income *(low (0); medium (1); high (2))*, nationality *(other nationality (0), German (1))*, region *(urban (0); semi-urban (1); rural (2))*. For less than two variables with p < 0.25, no model is calculated.

A pvalue less than 0.05 is considered statistically significant. Data are analysed using the software IBM Statistical Package for Social Sciences version 27 (SPSS Inc., Chicago, IL, USA).

## Results

### Sample characteristics

In the respective analyses, 5,160 children and adolescents each in IG and CG can be included for the U10, 5,861 each for the U11 and 1,808 each for the J2. Due to the matching, there are no differences with regard to age, gender, federal state or the presence of a chronic illness between IG and CG. The U10 sample has an average age of 7.7 years (SD: 0.5), the U11 of 10.0 years (SD: 0.7) and the J2 of 16.6 years (SD: 0.5). In all groups, the proportion of females is higher. 26.0% of the U10 sample, 29.8% of the U11 sample and 47.2% of the J2 sample have a chronic illness. Concerning the other determinants examined, i.e. those that are not matching criteria, there are differences between IG and CG. In all groups, the directly insured person (usually father or mother) in IG is more likely to have a higher level of school-leaving education and occupational education, and a higher income and is less likely to be unemployed compared to CG. The children and adolescents of IG live more often in urban regions and less often in rural regions. In terms of nationality, the proportion of children and adolescents with a nationality other than German is lower in IG for the U10 and the U11, while it is reversed with respect to the J2. The characteristics of the three samples stratified according to the different parameters as well as the significance levels are shown in Table 2.

**Table 2:** Sample characteristics

	U10			U11			J2		
	IG n = 5,160	CG n = 5,160	p	IG n = 5,861	CG n = 5,861	p	IG n = 1,808	CG n = 1,808	p
**Age** (Ø)	7.7	7.7	-	10.0	10.0	-	16.6	16.6	-
**Sex** (female)	55.0%	55.0%	-	54.2%	54.2%	-	49.7%	49.7%	-
**Federal State**			-			-			-
B	65.2%	65.2%		62.0%	62.0%		61.2%	61.2%	
BRB	20.5%	20.5%		22.2%	22.2%		21.8%	21.8%	
MV	14.4%	14.4%		15.8%	15.8%		17.0%	17.0%	
**Chronic disease** (yes)	26.0%	26.0	-	29.8%	29.8	-	47.2%	47.2%	-
**School leaving** qualification*			0.036			≤ 0.001			0.846
None or low	34.5%	36.1%		33.3%	37.1%		34.8%	36.1%	
Medium	45.9%	47.1%		48.5%	49.3%		51.7%	51.2%	
High	19.5%	16.8%		18.2%	13.6%		13.5%	12.7%	
*Missing*	*2,412*	*2,621*		*2,847*	*2,980*		*1,133*	*1,085*	
**Vocational** qualification*			0.046			0.004			0.403
None	22.9%	24.8%		21.7%	23.6%		20.2%	20.8%	
Completed vocational training	64.1%	64.1%		66.1%	66.7%		69.0%	70.4%	
Higher degrees**	13.0%	11.1%		12.2%	9.7%		10.8%	8.7%	
*Missing*	*2,384*	*2,600*		*2,784*	*2,915*		*1,094*	*1,050*	
**Insurance and** occupational status*			≤ 0.001			≤ 0.001			0.180
Compulsorily insured	58.6%	52.9%		58.8%	54.3%		58.3%	56.5%	
Voluntarily insured	4.8%	4.5%		5.8%	4.5%		6.8%	5.4%	
Unemployed	35.3%	41.1%		33.7%	38.8%		29.9%	33.1%	
Other	1.3%	1.4%		1.7%	2.4%		5.1%	5.0%	
*Missing*	*39*	*55*		*64*	*100*		*392*	*335*	
Income*			0.166			0.065			0.028
Low	59.0%	61.1%		56.8%	58.7%		53.5%	49.9%	
Medium	32.1%	31.1%		33.7%	33.2%		35.9%	41.6%	
High	8.9%	7.8%		9.5%	8.1%		10.6%	8.5%	
*Missing*	*1,920*	*2,211*		*2,104*	*2,444*		*864*	*867*	
**Nationality**			≤ 0.001			0.520			0.300
German	84.5%	81.1%		79.3%	78.8%		73.1%	74.6%	
Other	15.5%	18.9%		20.7%	21.2%		26.9%	25.4%	
*Missing*	*30*	*38*		*42*	*48*		*19*	*18*	
**Region**			≤ 0.001			≤ 0.001			≤ 0.001
Rural	11.3%	14.4%		11.4%	15.1%		9.6%	14.7%	
Semi-urban	16.1%	14.7%		18.3%	16.7%		18.3%	16.9%	
Urban	72.6%	70.9%		70.3%	68.2%		72.1%	68.3%	
*Missing*	*0*	*0*		*0*	*2*		*1*	*2*	

*Status of the directly insured person (normally father or mother); ** degrees from universities. higher education

### Establishments and technical colleges; p = pearson-chi-quadrat

**Hypothesis H**_**1**_. Overall, the proportion of children with an incident diagnosis differs significantly between IG and CG for the U10, U11 and J2. With respect to the U10 25.0% of IG have one of the defined diagnoses and 12.4% of CG (p ≤ 0.001). For the U11, the proportion differs between 29.7% in IG and 17.7% in CG (p ≤ 0.001) and for the J2, it differs between 16.7% in IG and 11.0% in CG (p ≤ 0.001). Across all diagnosis groups, IG shows a larger number of cases than CG, and the respective values are shown in Tables 3, 4 and 5.

**Hypothesis H**_**2**_. The average age at diagnosis shows a significant difference between IG and CG for all three examinations (see Tables 3, 4 and 5). In the U10, IG children are on average 8.3 years old and CG children are 8.9 years old (p ≤ 0.001). The mean age at diagnosis in the U11 is 10.2 years for IG and 10.8 years for CG (p ≤ 0.001), and in relation to the J2, adolescents in IG (16.9 years) are also significantly younger than those in CG (17.3 years) (p ≤ 0.001).

**Hypothesis H**_**3**_. The proportion of children who subsequently received therapy shows significant differences both for the overall groups as well as for the group for which a diagnosis was made for the U10 and U11. While, when considering the entire population, proportionally more children in IG received therapy in relation to CG, this trend is reversed for the group of those with a diagnosis in the post-observation period (see Tables 3 and 4). Considering the total sample, there is in the U10, for example, a difference of 9.1% in IG to 5.4% in CG, while considering those diagnosed, the values are 36.4% in IG and 43.3% in CG. For the J2 the overall proportion with therapy is also significantly higher in IG than in CG, while among those with a diagnosis, there is no significant difference, but the proportion in CG is still higher (see Table 5).

**Hypothesis H**_**4**_. For all three examinations the average age at the time of initiation of the therapy is significantly lower in IG than in CG (see Tables 3, 4 and 5). The children in IG of the U10 sample are on average 8.5 years old and those in CG 8.9 years old (p ≤ 0.001). The average age at initiation of therapy in the U11 was 10.4 years in IG and 10.9 years in CG (p ≤ 0.001) and in the J2 17.4 years in IG and 17.8 years in CG (p ≤ 0.001).

**Table 3:** U10: Diagnoses and therapies

	IG (n = 5,160)		CG (n = 5,160)		p-value
	n	Mean/Percent	n	Mean/Percent	
**Proportion with diagnosis**					
Overall	1,292	25.0%	642	12.4%	≤ 0.001^1^
*F80-F89*	*411*	*8.0%*	*174*	*3.4%*	
*F90-F98*	*562*	*10.9%*	*336*	*6.5%*	
*Both*	*319*	*6.2%*	*132*	*2.6%*	
**Average age at diagnosis**	1,292	8.3	642	8.9	≤ 0.001^2^
**Proportion with therapy**					
Overall	470/5,160	9.1%	278/5,160	5.4%	≤ 0.001^1^
Of diagnosed	470/1,292	36.4%	278/642	43.3%	0.003^1^
**Average age at therapy start**	470	8.5	278	8.9	≤ 0.001^2^

^1^ Fisher’s exact test; ^2^ Mann-Whitney U test

**Table 4:** U11: Diagnoses and therapies

	IG (n = 5,861)		CG (n = 5,861)		p-value
	n	Mean/Percent	n	Mean/Percent	
**Proportion with diagnosis**					
Overall	1,739	29.7%	1,038	17.7%	≤ 0.001^1^
*F19.0-F19.2*	*2*	*0.0%*	*1*	*0.0%*	
*F50; E66*	*30*	*0.5%*	*9*	*0.2%*	
*F80-F89; F90-F98*	*882*	*15.0%*	*504*	*8.6%*	
*F43.2; F45.3-F45.4*	*91*	*1.6%*	*92*	*1.6%*	
*F45.8; Z73*	449	*7.7%*	259	*4.4%*	
*K00-K14*	285	4.9%	173	3.0%	
*More than one diagnosis*					
**Average age at diagnosis**	1,739	10.2	1,038	10.8	≤ 0.001^2^
**Proportion with therapy**					
Of all	395/5,861	6.7%	329/5,861	5.6%	0.013^1^
Of diagnosed	395/1,739	22.7%	329/1,038	`31.7%	≤ 0.001^1^
**Average age at therapy start**	395	10.4	329	10.9	≤ 0.001^2^

^1^ Fisher’s exact test; ^2^ Mann-Whitney U test

**Table 5:** J2: Diagnoses and therapies

	IG (n = 1,808)		CG (n = 1,808)		p-value
	n	Mean/Percent	n	Mean/Percent	
**Proportion with diagnosis**					
*E00-E07*	302	16.7%	198	11.0%	≤ 0.001^1^
*E11-E14*	*83*	*4.6%*	*67*	*3.7%*	
*E30*	*8*	*0.4%*	*2*	*0.1%*	
*F52; F64; F65*	*1*	*0.1%*	*0*	*0.0%*	
*M40; M41; M43*	*14*	*0.8%*	*12*	*0.7%*	
*More than one diagnosis*	*172* *24*	*9.5%* *1.3%*	*104* *13*	*5.8%* *0.7%*	
**Average age at diagnosis**	302	16.9	198	17.3	≤ 0.001^2^
**Proportion with therapy**					
Of all	163/1,808	9.1%	118/1,808	6.5%	0.006^1^
Of diagnosed	163/302	53.9%	118/198	60.0%	0.232^1^
**Average age at therapy start**	163	17.4	118	17.8	≤ 0.001^2^

^1^ Fisher’s exact test; ^2^ Mann-Whitney U test

### Subgroup-specific analyses

For the U10 and U11, it is apparent across all subgroups that IG has a significantly higher proportion of children with a diagnosis than CG (see Table 6). However, there are subgroup-specific differences that apply to IG and CG. The detection rate in semi-urban regions is, for example, higher than that in rural and urban areas across all groups. The other determinants considered seem to have an influence on the detection rate, showing various strata-specific differences. For the U10, the rates for IG vary between 20.0% and 29.3% and for CG from 9.1–13.3%, while for the U11 they range from 26.0–35.7% in IG and from 12.3–20.6% in CG. With one exception, the detection rate of the J2 across all subgroups is also higher in IG than in CG. In relation to insurance and occupational status the rate in the combined category ‘other’ is higher in IG than in CG, but this difference is not significant. Non-significant differences were also found for some other strata. Significant differences by contrast are shown regarding compulsorily insured, unemployed, low and medium income groups, German nationality as well as semi-urban and urban regions (see Table 6). Overall, the rates in the J2 for IG vary between 12.1% and 20.8% and for CG from 7.5 to 16.2%.

**Table 6:** Proportions with defined diagnosis stratified by different parameters for U10, U11 and J2

	U10					U11					J2				
School leaving qualification*	IG (n = 2,748)		CG (n = 2,539)		p	IG (n = 3,014)		CG (n = 2,881)		p	IG (n = 675)		CG (n = 723)		p
None or low	248	26.1%	104	11.4%	≤ 0.001	307	30.6%	183	17.1%	≤ 0.001	37	15.7%	33	12.6%	0.366
Medium	319	25.3%	157	13.1%	≤ 0.001	461	31.6%	227	16.0%	≤ 0.001	58	16.6%	43	11.6%	0.068
High	110	20.5%	43	10.1%	≤ 0.001	143	26.0%	62	15.8%	≤ 0.001	11	12.1%	9	9.8%	0.644
Vocational qualification*	IG (n = 2,776)		CG (n = 2,560)		p	IG (n = 3,077)		CG (n = 2,946)		p	IG (n = 714)		CG (n = 758)		p
None	161	25.4%	70	11.0%	≤ 0.001	201	30.1%	122	17.6%	≤ 0.001	26	18.1%	21	13.3%	0.270
Completed vocational training	458	25.7%	198	12.1%	≤ 0.001	628	30.9%	311	15.8%	≤ 0.001	75	15.2%	61	11.4%	0.080
Higher degrees**	80	22.1%	36	12.7%	0.002	97	25.8%	49	17.1%	0.008	16	20.8%	6	9.1%	0.065
Insurance and occupational status*	IG (n = 5,121)		CG (n = 5,105)		p	IG (n = 5,797)		CG (n = 5,761)		p	IG (n = 1,416)		CG (n = 1,473)		p
Compulsorily insured	710	23.7%	329	12.2%	≤ 0.001	992	29.1%	491	15.7%	≤ 0.001	144	17.5%	94	11.3%	≤ 0.001
Voluntarily insured	62	25.3%	23	10.0%	≤ 0.001	99	29.4%	38	14.8%	≤ 0.001	12	12.5%	6	7.5%	0.325
Unemployed	495	27.4%	261	12.4%	≤ 0.001	588	30.1%	460	20.6%	≤ 0.001	71	16.8%	51	10.5%	0.006
Other	20	30.3%	9	12.3%	0.012	35	35.7%	28	20.3%	0.011	11	15.3%	12	16.2%	1.000
Income*	IG (n = 3,240)		CG (n = 2,949)		p	IG (n = 3,757)		CG (n = 3,417)		p	IG (n = 944)		CG (n = 941)		p
Low	467	24.4%	233	12.9%	≤ 0.001	622	29.1%	324	16.2%	≤ 0.001	85	16.8%	52	11.1%	0.010
Medium	234	22.5%	100	10.9%	≤ 0.001	380	30.0%	182	16.0%	≤ 0.001	61	18.0%	42	10.7%	0.006
High	68	23.7%	21	9.1%	≤ 0.001	101	28.2%	34	12.3%	≤ 0.001	20	20.0%	9	11.3%	0.153
**Nationality**	IG (n = 5,130)		CG (n = 5,122)		p	IG (n = 5,819)		CG (n = 5,813)		p	IG (n = 1,789)		CG (n = 1,790)		p
German	1,122	25.9%	527	12.7%	≤ 0.001	1410	30.6%	813	17.7%	≤ 0.001	221	16.9%	140	10.5%	≤ 0.001
Other	159	20.0%	112	11.6%	≤ 0.001	316	26.2%	215	17.5%	≤ 0.001	76	15.8%	57	12.6%	0.161
**Region**	IG (n = 5,160)		CG (n = 5,160)		p	IG (n = 5,861)		CG (n = 5,859)		p	IG (n = 1,807)		CG (n = 1,806)		p
Rural	139	23.9%	84	11.3%	≤ 0.001	198	29.6%	137	15.5%	≤ 0.001	30	17.3%	30	11.3%	0.088
Semi-urban	244	29.3%	101	13.3%	≤ 0.001	366	34.2%	182	18.6%	≤ 0.001	55	16.7%	30	9.8%	0.014
Urban	909	24.3%	457	12.5%	≤ 0.001	1175	28.5%	718	18.0%	≤ 0.001	217	16.6%	137	11.1%	≤ 0.001

*Status of the directly insured person (normally father or mother); ** degrees from universities. higher education establishments and technical colleges, p = Fisher’s exact test

### Regression analyses

For the U10, U11 and J2, regression models show that participation in the examinations has the strongest influence on detecting a diagnosis among the included variables. The presence of a chronic disease and sex - for the U10 and U11 males and for the J2 females - also tend to more often predict the detection of a diagnosis. The model for the U10 further shows that German nationality and unemployment have a significant effect. In the model for the U11, German nationality, unemployment and living in a semi-rural region are associated with detecting a diagnosis (see Table 7). The disease-specific models for the U11 (1. diseases of oral cavity, salivary glands and jaws; 2. mental and behavioural disorders) and the J2 (1. deforming dorsopathies; 2. endocrine diseases) consistently show that participation in examinations is a significant predictor for the detection of a diagnosis (see appendix, table A1.1-A1.5). Considering the initiation of treatment in overall samples, the models show similar results, but the presence of a chronic illness has the highest predictive power for all three examinations. For the U10 and J2, participation in the examinations as well as for the U11 male and the J2 female sex tend to more often predict treatment initiation, while for the U11 treatment initiation is additionally associated with German nationality and unemployment (see Table 8). Regarding the initiation of treatment in diagnosed individuals, only a model for the U11 can be calculated based on the requirements for regression analyses. The model shows that initiation of therapy is significantly correlated with non-participation in the examinations, presence of a chronic disease and German nationality (see appendix, table A2.1-A2.2).

**Table 7:** Binominal logistic regression analyses for diagnosis within U10, U11 and J2 examinations (H_1_)

**U10***(n = 10,158; missing: 162; HL-Test χ² (8) = 14.230, p > 0,05; Nagelkerke’s R*^*2*^ *= 0.088)*									
		**RegB**	**SD**	**Wald**	**df**	**Sig.**	**Exp(B)**	**95%-CI**	
AOK-Junior	Yes vs. no *(Ref.)*	0.899	0.055	269.332	1	< 0.001	2.458	2.208	2.737
Sex	Male vs. female *(Ref.)*	0.381	0.053	52.521	1	< 0.001	1.464	1.320	1.622
Chronic disease	Yes vs. no *(Ref.)*	0.782	0.055	200.638	1	< 0.001	2.186	1.962	2.436
Nationality	German vs. other nationality *(Ref.)*	0.250	0.076	10.880	1	0.001	1.284	1.107	1.489
Unem-ployment	Yes vs. no (Ref.)	0.156	0.054	8.196	1	0.004	1.169	1.050	1.301
Region	Urban *(Ref.)*			6.520	2	0.038			
	Semi-urban	0.134	0.072	3.475	1	0.062	1.144	0.993	1.317
	Rural	-0.113	0.084	1.789	1	0.181	0.894	0.758	1.054
Constant		-2.577	0.083	969.362	1	< 0.001	0.076		
**U11***(n = 11,470; missing: 252; HL-Test χ² (8) = 8.916, p > 0,05; Nagelkerke’s R*^*2*^ *= 0.046)*									
		**RegB**	**SD**	**Wald**	**df**	**Sig.**	**Exp(B)**	**95%-CI**	
AOK-Junior	Yes vs. no (Ref.)	0.685	0.046	225.644	1	< 0.001	1.984	1.814	2.170
Sex	Male vs. female (Ref.)	0.189	0.045	17.780	1	< 0.001	1.208	1.106	1.318
Chronic disease	Yes vs. no (Ref.)	0.414	0.047	76.835	1	< 0.001	1.512	1.379	1.659
Nationality	German vs. other nationality (Ref.)	0.134	0.058	5.218	1	0.022	1.143	1.019	1.282
Unem-ployment	Yes vs. no (Ref.)	0.631	0.314	4.043	1	0.044	1.879	1.016	3.476
Region	Urban (Ref.)			8.894	2	0.012			
	Semi-urban	0.149	0.060	6.213	1	0.013	1.161	1.032	1.305
	Rural	-0.072	0.071	1.017	1	0.313	0.931	0.809	1.070
Constant		-1.969	0.068	830.715	1	< 0.001	0.140		
**J2***(n = 3,616; missing: 0; HL-Test χ² (6) = 7.352, p > 0,05; Nagelkerke’s R*^*2*^ *= 0.036)*									
		**RegB**	**SD**	**Wald**	**df**	**Sig.**	**Exp(B)**	**95%-CI**	
AOK-Junior	Yes vs. no (Ref.)	0.495	0.099	25.11	1	< 0.001	1.641	1.352	1.992
Sex	Male vs. female (Ref.)	-0.497	0.099	25.238	1	< 0.001	0.609	0.501	0.739
Chronic disease	Yes vs. no (Ref.)	0.442	0.098	20.407	1	< 0.001	1.556	1.284	1.885
Constant		-2.099	0.102	423.735	1	< 0.001	0.123		

*Ref.=reference; RegB = regression coefficient; SD = standard deviation; Wald = test statistic; df = degrees of freedom; Sig.=significance level; Exp(B) = odds ratio; 95%-CI = confidence interval; HL-Test = Hosmer–Lemeshow test*

**Table 8:** Binominal logistic regression analyses for treatment initiation (H_3_)

**U10***(n = 10,252; missing: 68; HL-Test χ² (7) = 7.668, p > 0,05; Nagelkerke’s R*^*2*^ *= 0.038)*									
		**RegB**	**SD**	**Wald**	**df**	**Sig.**	**Exp(B)**	**95%-CI**	
AOK-Junior	Yes vs. no *(Ref.)*	0.564	0.079	50.958	1	< 0.001	1.758	1.506	2.053
Sex	Male vs. female *(Ref.)*	0.405	0.077	27.615	1	< 0.001	1.500	1.289	1.745
Chronic disease	Yes vs. no *(Ref.)*	0.688	0.079	75.889	1	< 0.001	1.989	1.704	2.322
Nationality	German vs. other nationality *(Ref.)*	-0.124	0.107	1.362	1	0.243	0.883	0.717	1.088
Constant		-3.266	0.082	1568.237	1	< 0.001	0.038		
**U11***(n = 11,470; missing: 252; HL-Test χ² (8) = 7.745, p > 0,05; Nagelkerke’s R*^*2*^ *= 0.018)*									
		**RegB**	**SD**	**Wald**	**df**	**Sig.**	**Exp(B)**	**95%-CI**	
AOK-Junior	Yes vs. no (Ref.)	0.222	0.078	8.011	1	0.005	1.249	1.071	1.456
Sex	Male vs. female (Ref.)	0.220	0.078	7.968	1	0.005	1.246	1.070	1.452
Chronic disease	Yes vs. no (Ref.)	0.529	0.080	44.300	1	< 0.001	1.698	1.453	1.984
Nationality	German vs. other nationality (Ref.)	0.309	0.104	8.856	1	0.003	1.362	1.111	1.670
Unem-ployment	Yes vs. no (Ref.)	0.263	0.080	10.871	1	0.001	1.301	1.112	1.521
Constant		-3.483	0.123	806.205	1	< 0.001	0.031		
**J2***(n = 3,616; missing: 0; HL-Test χ² (6) = 6.399, p > 0,05; Nagelkerke’s R*^*2*^ *= 0.035)*									
		**RegB**	**SD**	**Wald**	**df**	**Sig.**	**Exp(B)**	**95%-CI**	
AOK-Junior	Yes vs. no (Ref.)	0.373	0.127	8.614	1	0.003	1.452	1.132	1.863
Sex	Male vs. female (Ref.)	-0.649	0.130	24.813	1	< 0.001	0.523	0.405	0.675
Chronic disease	Yes vs. no (Ref.)	0.544	0.128	18.156	1	< 0.001	1.722	1.341	2.212
Constant		-2.689	0.132	417.248	1	< 0.001	0.068		

*Ref.=reference; RegB = regression coefficient; SD = standard deviation; Wald = test statistic; df = degrees of freedom; Sig.=significance level; Exp(B) = odds ratio; 95%-CI = confidence interval; HL-Test = Hosmer–Lemeshow test*

## Discussion

### Hypotheses

Hypothesis H_1_ (More diseases are detected in IG than in CG.) is confirmed. The screening modules U10, U11 and J2 show that the diseases that the respective screening is supposed to detect are detected more frequently in IG than in CG. In this context, the hypothesis H_2_ (Diseases are detected earlier in IG than in CG.) and H_4_ (Early detection of disease in IG leads to earlier therapy than in CG.) can subsequently be confirmed as statistically significant. The analyses show that diseases are detected slightly earlier in IG than in CG and, in the case of therapy, it is also initiated slightly earlier. A review indicates that early initiation of therapy is associated with better health outcomes later in life, e.g. early speech therapy partially improves expressive language skills [19, 20]. Since the difference between the groups in our analyses is always only about half a year, it must be examined over a longer time horizon to what extent the statistically significant age differences found are also clinically significant. Concerning H_3_ (Early detection of disease in IG leads to more therapies than in CG.), it is discovered that among children and adolescents with a diagnosis, fewer therapeutic services are billed by physicians in IG than in CG. One reason for this can be the recognition of early stages of the disease, for which no specific therapy is indicated. The diagnosis of pre- or early stages is a goal of the early detection examinations and a corresponding therapy should only be initiated if necessary [21–23]. This raises the question of the relevance of early diagnosis without initiation of therapy. One advantage is that the paediatrician may be aware that the disease may develop further, so that he or she can monitor it and thereby prevent it from progressing or manifesting, while children and adolescents in CG are diagnosed later by symptomatic presentation and must be treated directly. In addition, IG has a higher rate in terms of the proportion of the total group receiving therapy, so that if one assumes equal prevalence in both groups, more children and adolescents with the disease are already diagnosed and receiving treatment in IG. However, it should be taken into account that overdiagnoses are sometimes made, especially in the area of mental and behavioural disorders in childhood and adolescence, e.g. for ADHD or developmental disorders, as studies have shown [24–26].

### U10 and U11

 With regard to the U10 and U11, it becomes obvious that, among the examined diagnoses, mainly incident diagnoses for disorders of psychological development and behavioural and emotional disorders appear in the post-observation period. Concerning the U10, the proportion of children with such a diagnosis in IG is above the average for this age group in Germany, while the proportion in CG is lower compared to the average for this age group [27]. At the U11, the proportion of children with diagnoses for disorders of psychological development and behavioural and emotional disorders is comparatively lower than the average for this age group in Germany in both IG and CG [27]. Regarding the values that are below the average, it should be considered that in the context of our analyses only incident cases after screening were considered, so previously diagnosed cases are not included. It can therefore be assumed that the value in the overall population is higher. Furthermore, it should be noted that, with respect to diagnoses examined in both screenings (F80-F89; F90-F98), children who participated in the U10 and U11 may have already been diagnosed in the U10 and are hence excluded in the analysis for the U11, as they are no longer incident cases.

### J2

Spinal disorders (kyphosis and lordosis, scoliosis, other deforming dorsopathies) and disorders of thyroid gland are the most common diagnoses of those analysed, while diabetes mellitus type II, disorders of puberty as well as sexual dysfunction, gender identity disorders and disorders of sexual preference seem to play a minor role in this age group. Concerning somatic diseases, this order is consistent with the overall population in Germany [28, 29]. The high proportion of adolescents with a chronic illness (47.2%) who took part in the J2 deviates strongly from the average value for this age group [29, 30]. The fact that there are more visits to the doctor due to the chronic illness could influence the higher attendance rate among the chronically ill individuals. It should be noted that the rate of participation in early detection examinations decreases with increasing age in the overall population [3, 22]. However, studies indicate that during adolescence, health-related behaviours change systematically and risky behaviours can develop [31]. Therefore, adolescence is an important period for prevention. Despite this, studies have concluded that often only small effects can be achieved for adolescents in this area. Large effects, on the other hand, can only be achieved for defined subgroups. This could be an indication that screening in adolescence may need to be more targeted. For most interventions, there has not yet been systematic research on which strategies are most effective for which age group [31].

### Subgroups

While screening is effective across almost all subgroups and IG had a higher detection rate compared to CG, there are differences between the individual strata, e.g. with regard to the region in which the children and adolescents live, their nationality or socio-economic parameters. One reason for this may be different lifestyles of the parents. Thus, socio-economic parameters, such as the education of the parents and their income, or the migration background, appear to have an influence on health behaviours (e.g. physical activity, nutrition) and thus consequently on the development of lifestyle-related diseases. This could also be shown in a Germany-wide survey: children and adolescents with a low socio-economic status are more likely to eat unhealthily, do less sports and are more likely to be overweight or obese than those from families with a higher socio-economic status [32]. This trend is also evident in respect to mental health problems: Children and adolescents from families with a low socio-economic status are considerably more likely to have mental health problems than their age group from families with a medium and high socio-economic status [27].

### Regression analyses

The regression analyses consistently show that participation in the U10, U11 or J2 more often predicts the detection of a diagnosis, and for the total sample also predicts the initiation of a therapy. Furthermore, sex, the presence of a chronic illness, nationality, unemployment, and region partly show a significant influence. The fact that male children are more likely to receive a diagnosis in the U10 and U11 is likely due to mental and behavioural disorders. A nationwide German survey also shows higher prevalences in boys [27, 32]. Regarding the results of the regression for the J2, studies show similar results for the influence of sex, e.g., that diseases of the thyroid gland occurred more frequently in girls [28]. Furthermore, nationality seems to have an influence on certain diagnoses, but comparability is difficult because most studies use the exact migration background. In those studies, it appears that the migration background has an influence on different health outcomes [33]. In terms of region of residence, contrary to our results, a longitudinal analysis of nationwide physician billing data shows that there are no differences in the prevalence of mental illness between urban and rural areas [34]. Concerning socio-economic determinants, studies indicate that psychosocial stress in parents, e.g. due to unemployment, is a risk factor for emotional and behavioural problems in children and adolescents [35, 36]. This is consistent with the results of the disease-specific regression for the U11. School-leaving and occupational qualifications as well as income do not appear to have a significant influence on either outcome in our analyses. This is partially in contrast to other studies that have found an association between, inter alia, mental illness and socio-economic status, as mentioned above, and the U10 and U11 focus precisely on these diseases [27, 37].

### International findings

Other countries, such as the United States, Australia, Canada, Belgium, Scotland and Austria, also recommend and offer different early detection examinations for different age groups in childhood and adolescence [38–42]. One example is the American Academy of Pediatrics Recommendations for Preventive Pediatric Health Care, which includes very comprehensive recommendations from birth to the age of 21 and represents a complete preventive care over the entire period of childhood and adolescence [42]. Various studies in other countries have also shown that screening examinations in childhood and adolescence are effective for the detection of various diseases [43–45]. However, complaints about poor data on the effectiveness of early detection examinations, especially with regard to long-term results also exist in other countries. Results from long-term studies are particularly important in this context in order to be able to show the effects into adulthood, for example concerning diseases that often only manifest in middle to old age. In most studies, however, the follow-up periods in this context are too short [16, 46]. Another difficulty is the comparability of findings between different countries, as the services offered differ considerably and, if the related outcomes are assessed, different measures are often used [47, 48].

### Limitations

*AOK-Junior* is an existing programme in which insured individuals can enrol voluntarily since 2007, so that it cannot be evaluated by means of a randomised controlled trial. Therefore, known limitations exist with regard to the validity of the results [49]. One of the main strengths of the analyses is that it was possible to include very large numbers of cases. In the course of the analyses, care was taken to select a control group with as many identical characteristics as possible for each module by means of individual matching in a ratio of 1:1 with the aim of being able to attribute the observed differences between IG and CG. However, a bias due to non-recorded factors cannot be completely ruled out. However, the results cannot be generalised to all children and adolescents in Germany, as about one tenth of children and adolescents in Germany are privately insured and these, for example, have a higher socioeconomic status and lower morbidity on average than children and adolescents with statutory insurance [50]. Another limitation is that although it could be observed that diagnoses are made slightly earlier and therapies are initiated slightly earlier, what consequences this has for children and adolescents in the medium to long term (e.g. clinical outcomes) could not be analysed due to the time horizon and would have to be investigated in a long-term study. However, it is important for the further investigation of the effectiveness that the programme has led to more diseases being diagnosed and thus to a larger proportion of children having a chance of initiating treatment.

In addition, the generally known methodological and content-related limitations of claims data analyses must be considered [51]. However, studies have also shown that the quality of claims data has improved in recent years [52]. In relation to the interpretation of the results from the claims data analysis, it should be noted that the data were not specifically collected for the questions, but represent diagnoses and therapies documented by physicians for the billing of their services with the health insurance funds, which cannot be clinically tested and are thus limited in their validity. A systematic review of international studies of routine data on mental disorders indicates this [53]. In addition, the recording of diagnoses is dependent not only on the coding behaviour of physicians, but also on other factors such as ICD changes [54]. In this context, since the quality of inpatient data is considered high due to the DRG system (Diagnosis Related Groups) and that of outpatient data is considered less high, only confirmed diagnoses are considered in the analysis of outpatient data [55]. In addition, certain treatments cannot be included in the analyses of therapies because they are not part of standard care in Germany. This applies, for example, to learning therapies or nutritional counselling and exercise programmes.

Another limitation is that for some parameters (e.g. school-leaving qualifications or income) only the data for the directly insured person (normally father or mother) are available, so that, for example, only the socio-economic characteristics of one parent are taken into account. In addition, only the nationality of the child or adolescent can be considered and not the actual migration background. Furthermore, due to the number of cases, the nationality is divided only into German or other nationalities, despite the heterogeneity of the nationalities, so that these results are only transferable to a limited extent. Overall, the regression models show that the variables included contribute only partially to variance explanation, so it can be assumed that a number of other factors play a role in the detection of a diagnosis and initiation of treatment, inter alia socio-demographic parameters such as the living space.

However, in the course of the analyses, care was taken to ensure that the results could be reproduced, i.e. that the analyses were as valid and reliable as possible. In addition, attention was paid to detailed documentation throughout the evaluation, so that a high degree of transparency is ensured.

## Conclusions

The U10 and U11 examinations are important to close the gap in continuous medical care between the U9 at approximately age 5 and the first youth examination J1 at approximately age 13. Thus, screening is extended into the primary school age of the children and a continuous transition to the early detection examinations for adolescents is created. The J2 is especially important to close the gap between the J1 and adulthood; this can, inter alia, facilitate the transition to adult medicine. The results confirm that all three examinations lead to a significantly higher detection of diseases and result in a slightly earlier diagnosis and initiation of therapy. Furthermore, the present study indicates that the U10 and U11 are effective in terms of detection of diseases across all subgroups, i.e. irrespective of socio-economic status, nationality and region, and a higher number of diseases are diagnosed in IG than in CG for all strata examined. The same applies, with one exception, to the J2. The logistic regressions show that participation in the examinations has a significant influence on the detection of a diagnosis and the initiation of therapy, even when other factors are taken into account. The results on treatment initiation suggest that mild cases or early stages that do not yet require treatment are detected during screening; severe cases that already show symptoms are also detected without screening and subsequently treated. Notably, early diagnosis enables monitoring by the treating paediatrician, so that a disease may not develop or manifest itself at all.

However, the costs for the U10, U11 and J2 examinations are not yet reimbursed by all health insurance funds and are not part of the standard care in Germany. One reason for this is that their effectiveness has not yet been adequately evaluated [3, 16]. For this reason, the present results, which partially confirm the effectiveness of the examinations, represent an important contribution to the creation of an evidence base for these screening measures in children and adolescents. It has been shown that the U10, U11 and J2 examinations contribute to the diagnosis of age-specific diseases and thus enable the possibility of early treatment if necessary. In the future, it would be useful to investigate the U10, U11 and J2 examinations over a longer time horizon to establish whether slightly earlier diagnosis and slightly earlier initiation of therapy also have a clinically meaningful impact and lead to the prevention of the manifestation or progression of the diagnosed diseases and to the avoidance of secondary diseases. In particular, it should be investigated whether the diseases being screened for are the relevant ones, i.e. whether some diseases may be irrelevant for the age groups being addressed.

## Electronic supplementary material

Below is the link to the electronic supplementary material.

Supplementary Material 1

## Data Availability

The datasets analysed were used under licence for the current study. According to data protection and confidentiality clauses in the licence agreement the data is not publicly available. Data are however available from the authors upon reasonable request and with permission of AOK Nordost.

## Abbreviations

ADHD: Attention Deficit Hyperactivity Disorder
AOK: Allgemeine Ortskrankenkasse (general health insurance scheme)
CG: Control group
DRG: Diagnosis related groups
EPIVA: Evaluation of the Pediatric-Centered Integrated Care *AOK-Junior*
H: Hypothesis
ICD: International Statistical Classification of Diseases and Related Health Problems
IG: Intervention group
J2: Early detection examinations for adolescents aged 16–17 years
U10: Early detection examinations for children aged 7–8 years
U11: Early detection examinations for children aged 9–10 years
