# Supplementary material for: The effectiveness of additional screening examinations for children and adolescents in Germany: a longitudinal retrospective cohort study
Source: BMC Pediatr. 2023 Apr 11;23:164. doi: 10.1186/s12887-023-03988-1 (PMC10088104; doi:10.1186/s12887-023-03988-1)
Supplement: Supplementary file 1 — Supplementary Material 1 [file 12887_2023_3988_MOESM1_ESM.docx]

**Appendix**

**A1. Regression analyses for diagnosis within the U11 and J2 examinations (H_1_) for stratified by diagnosis groups.**

Binomial logistic regression modelling for H_1_ was performed stratified by diagnostic group to analyse whether the results obtained for the U11 and J2 applied to all diagnostic groups.

The following variables were first analysed and those shown to be associated with the outcome below p<0.25 were included in the respective models: group (IG/CG), sex (male/female), chronic disease (yes/no), school leaving qualification (none or low/medium/high), vocational qualification (none/completed vocational training/higher degrees), unemployment (yes/no), income (low/medium/high), nationality (German/other nationality), region (rural/semi-urban/urban).

Table A1.1: Test for association (Fisher's exact test) of parameters with diagnosis (yes/no) stratified by disease and examination.

| **Parameter** | AOK-Junior | Sex | Chron. Disease | School leaving qualification | Vocational qualification | Unemployment | Income | Nationality | Region |
| --- | --- | --- | --- | --- | --- | --- | --- | --- | --- |
| **U11** |  |  |  |  |  |  |  |  |  |
| Diseases of oral cavity, salivary glands and jaws (K00-K14) | <0.001 | 0.452 | 0.822 | 0.761 | 0.930 | 0.670 | 0.447 | 0.503 | 0.002 |
| Mental and behavioural disorders* | <0.001 | <0.001 | <0.001 | 0.405 | 0.374 | 0.002 | 0.296 | 0.002 | 0.001 |
| **J2** |  |  |  |  |  |  |  |  |  |
| Deforming dorsopathies (M40; M41; M43) | <0.001 | 0.338 | <0.001 | 0.624 | 0.519 | 0.747 | 0.897 | 0.583 | 0.501 |
| Endocrine diseases (E00-E07; E11-E14; E30) | 0.015 | 0.012 | <0.001 | 0.387 | 0.792 | 0.429 | 0.356 | 0.029 | 0.144 |

*(F19.0-F19.2; F50; E66; F80-F89; F90-F98; F43.2; F45.3-F45.4; F45.8; Z73)

Table A1.2: Regression analyses for diagnosis of diseases of oral cavity, salivary glands and jaws (K00-K14 )within the U11.

|  |  | **RegB** | **SD** | **Wald** | **df** | **Sig.** | **Exp(B)** | **95%-CI** | |
| --- | --- | --- | --- | --- | --- | --- | --- | --- | --- |
| AOK-Junior | Yes vs. no *(Ref.)* | 0.642 | 0.071 | 81.57 | 1 | <0.001 | 1.900 | 1.653 | 2.184 |
| Region | Urban *(Ref.)* |  |  | 10.097 | 2 | 0.006 |  |  |  |
|  | Semi-urban | 0.219 | 0.086 | 6.544 | 1 | 0.011 | 1.245 | 1.053 | 1.473 |
|  | Rural | -0.155 | 0.111 | 1.949 | 1 | 0.163 | 0.856 | 0.688 | 1.065 |
| Constant |  | -2.831 | 0.061 | 2119.418 | 1 | <0.001 | 0.059 |  |  |
| *n= 11,720; missing: n=2; HL-Test χ² (4) = 4.480, p>0,05; Nagelkerke’s R^2^=0.019* | | | | | | | | | |

*Ref.=reference; RegB=regression coefficient; SD=standard deviation; Wald=test statistic; df=degrees of freedom; Sig.=significance level; Exp(B)=odds ratio; 95%-CI=confidence interval; HL-Test=* *Hosmer–Lemeshow test*

Table A1.3: Regression analyses for diagnosis of mental and behavioural disorders (F19.0-F19.2; F50; E66; F80-F89; F90-F98; F43.2; F45.3-F45.4; F45.8; Z73) within the U11.

|  |  | **RegB** | **SD** | **Wald** | **df** | **Sig.** | **Exp(B)** | **95%-CI** | |
| --- | --- | --- | --- | --- | --- | --- | --- | --- | --- |
| AOK-Junior | Yes vs. no *(Ref.)* | 0.628 | 0.051 | 151.197 | 1 | <0.001 | 1.874 | 1.696 | 2.071 |
| Sex | Male vs. female *(Ref.)* | 0.267 | 0.050 | 28.550 | 1 | <0.001 | 1.306 | 1.184 | 1.440 |
| Chronic disease | Yes vs. no *(Ref.)* | 0.555 | 0.052 | 115.350 | 1 | <0.001 | 1.742 | 1.574 | 1.927 |
| Nationality | German vs. other nationality *(Ref.)* | 0.187 | 0.066 | 7.925 | 1 | 0.005 | 1.205 | 1.058 | 1.372 |
| Region | Urban *(Ref.)* |  |  | 7.151 | 2 | 0.028 |  |  |  |
|  | Semi-urban | 0.161 | 0.066 | 5.907 | 1 | 0.015 | 1.174 | 1.032 | 1.336 |
|  | Rural | -0.040 | 0.079 | 0.258 | 1 | 0.612 | 0.960 | 0.822 | 1.122 |
| Unem-ployment | Yes vs. no *(Ref.)* | 0.222 | 0.052 | 18.202 | 1 | <0.001 | 1.249 | 1.128 | 1.383 |
| Constant |  | -2.468 | 0.077 | 1016.130 | 1 | <0.001 | 0.085 |  |  |
| *n= 11,470; missing: n=252; HL-Test χ² (8) = 6.718, p>0,05; Nagelkerke’s R^2^=0.048* | | | | | | | | | |

*Ref.=reference; RegB=regression coefficient; SD=standard deviation; Wald=test statistic; df=degrees of freedom; Sig.=significance level; Exp(B)=odds ratio; 95%-CI=confidence interval; HL-Test=* *Hosmer–Lemeshow test*

Table A1.4: Regression analyses for diagnosis of deforming dorsopathies (M40; M41; M43) within the J2.

|  |  | **RegB** | **SD** | **Wald** | **df** | **Sig.** | **Exp(B)** | **95%-CI** | |
| --- | --- | --- | --- | --- | --- | --- | --- | --- | --- |
| AOK-Junior | Yes vs. no *(Ref.)* | 0.596 | 0.124 | 23.093 | 1 | <0.001 | 1.815 | 1.424 | 2.315 |
| Chronic disease | Yes vs. no *(Ref.)* | 0.540 | 0.122 | 19.548 | 1 | <0.001 | 1.716 | 1.350 | 2.179 |
| Constant |  | -3.004 | 0.121 | 612.623 | 1 | <0.001 | 0.05 |  |  |

*n= 3,616; missing: n=0; HL-Test χ² (2) = 1.077. p>0.05; Nagelkerke’s R^2^=0.027*

*Ref.=reference; RegB=regression coefficient; SD=standard deviation; Wald=test statistic; df=degrees of freedom; Sig.=significance level; Exp(B)=odds ratio; 95%-CI=confidence interval; HL-Test=* *Hosmer–Lemeshow test*

Table A1.5: Regression analyses for diagnosis of endocrine diseases (E00-E07; E11-E14; E30) within the J2.

|  |  | **RegB** | **SD** | **Wald** | **df** | **Sig.** | **Exp(B)** | **95%-CI** | |
| --- | --- | --- | --- | --- | --- | --- | --- | --- | --- |
| AOK-Junior | Yes vs. no *(Ref.)* | 0.360 | 0.151 | 5.648 | 1 | 0.017 | 1.433 | 1.065 | 1.928 |
| Sex | Male vs. female *(Ref.)* | -0.960 | 0.163 | 34.861 | 1 | <0.001 | 0.383 | 0.278 | 0.527 |
| Chronic disease | Yes vs. no *(Ref.)* | 0.416 | 0.151 | 7.539 | 1 | 0.006 | 1.516 | 1.126 | 2.039 |
| Nationality | German vs. other nationality *(Ref.)* | -0.342 | 0.170 | 4.038 | 1 | 0.044 | 0.711 | 0.509 | 0.992 |
| Region | Urban *(Ref.)* |  |  | 1.680 | 2 | 0.432 |  |  |  |
|  | Semi-urban | -0.128 | 0.214 | 0.357 | 1 | 0.550 | 0.880 | 0.579 | 1.338 |
|  | Rural | -0.347 | 0.279 | 1.547 | 1 | 0.214 | 0.707 | 0.409 | 1.221 |
| Constant |  | -2.587 | 0.181 | 204.735 | 1 | <0.001 | 0.075 |  |  |
| *n= 3,579; missing: n=37; HL-Test χ² (8) = 3.578. p>0.05; Nagelkerke’s R^2^=0.048*  *Ref.=reference; RegB=regression coefficient; SD=standard deviation; Wald=test statistic; df=degrees of freedom; Sig.=significance level; Exp(B)=odds ratio; 95%-CI=confidence interval; HL-Test=* *Hosmer–Lemeshow test* | | | | | | | | | |

**A2. Regression analyses for treatment initiation (H_3_) for the group with prior diagnosis.**

Additional binomial logistic regression modelling for H_3_ was performed for the subgroup with prior diagnosis.

The following variables were first analysed and those shown to be associated with the outcome below p<0.25 were included in the respective models: group (IG/CG), sex (male/female), chronic disease (yes/no), school leaving qualification (none or low/medium/high), vocational qualification (none/completed vocational training/higher degrees), unemployment (yes/no), income (low/medium/high), nationality (German/other nationality), region (rural/semi-urban/urban). For less than two variables with p<0.25, no model was calculated. For the U10 and J2 no model was calculated because the conditions for a regression were not given.

Table A2.1: Test for association (Fisher's exact test) of parameters with treatment (yes/no) stratified by examination for the subgroup with prior diagnosis.

| **Parameter** | AOK-Junior | Sex | Chron. Disease | School leaving qualification | Vocational qualification | Unemployment | Income | Nationality | Region |
| --- | --- | --- | --- | --- | --- | --- | --- | --- | --- |
| **U10**  (IG: n=1.292;  CG: n=642) | 0.028 | 0.103 | 0.376 | 0.292 | 0.780 | 0.784 | 0.398 | 0.410 | 0.758 |
| **U11**  (IG: n=1.793;  CG: n=1.038) | <0.001 | 0.185 | 0.003 | 0.662 | 0.369 | 0.066 | 0.288 | 0.039 | 0.003 |
| **J2**  (IG: n=302;  CG: n=198) | 0.310 | 0.034 | 0.121 | 0.382 | 0.610 | 0.635 | 0.599 | 0.338 | 0.914 |

Table A2.2: Regression analyses for treatment initiation for the group with prior diagnosis in the U11.

|  |  | **RegB** | **SD** | **Wald** | **df** | **Sig.** | **Exp(B)** | **95%-CI** | |
| --- | --- | --- | --- | --- | --- | --- | --- | --- | --- |
| AOK-Junior | Yes vs. no *(Ref.)* | -0.410 | 0.090 | 20.721 | 1 | <0.001 | 0.663 | 0.556 | 0.792 |
| Sex | Male vs. female *(Ref.)* | 0.102 | 0.089 | 1.335 | 1 | 0.248 | 1.108 | 0.931 | 1.318 |
| Chronic disease | Yes vs. no *(Ref.)* | 0.235 | 0.091 | 6.747 | 1 | 0.009 | 1.265 | 1.059 | 1.511 |
| Nationality | German vs. other nationality *(Ref.)* | 0.258 | 0.121 | 4.578 | 1 | 0.032 | 1.295 | 1.022 | 1.640 |
| Region | Urban *(Ref.)* |  |  | 8.788 | 2 | 0.012 |  |  |  |
|  | Semi-urban | -0.224 | 0.120 | 3.505 | 1 | 0.061 | 0.799 | 0.632 | 1.011 |
|  | Rural | 0.244 | 0.135 | 3.268 | 1 | 0.071 | 1.276 | 0.980 | 1.662 |
| Unem-ployment | Yes vs. no *(Ref.)* | 0.127 | 0.091 | 1.936 | 1 | 0.164 | 1.135 | 0.949 | 1.358 |
| Constant |  | -1.184 | 139 | 72.265 | 1 | <0.001 | 0.306 |  |  |
| *n=2,709; missing: n=122; HL-Test χ² (8) = 5.109, p>0,05; Nagelkerke’s R^2^=0.025* | | | | | | | | | |

*Ref.=reference; RegB=regression coefficient; SD=standard deviation; Wald=test statistic; df=degrees of freedom; Sig.=significance level; Exp(B)=odds ratio; 95%-CI=confidence interval; HL-Test=* *Hosmer–Lemeshow test*
